# Supplementary material for: Global hypo-methylation in a proportion of glioblastoma enriched for an astrocytic signature is associated with increased invasion and altered immune landscape
Source: eLife. 2022 Nov 22;11:e77335. doi: 10.7554/eLife.77335 (PMC9681209; doi:10.7554/eLife.77335)
Supplement: Figure 2—source data 1. [file elife-77335-fig2-data1.zip › Figure_2_source_data_1/Figure_2C/homerResults/motif30.similar.html]

motif30

## Information for motif30

A
G
C
T
G
T
A
C
G
T
A
C
A
G
T
C
G
A
C
T
A
G
T
C
T
A
C
G
T
C
A
G
C
A
T
G
A
T
C
G
C
A
G
T
A
T
C
G
G
T
A
C
T
A
C
G
A
G
T
C
A
G
T
C
G
A
T
C
G
C
A
T
C
A
G
T
A
G
T
C
  
Reverse Opposite:  

C
T
A
G
G
T
C
A
C
G
T
A
C
T
A
G
A
C
T
G
T
C
A
G
A
T
G
C
C
A
T
G
A
T
G
C
G
T
C
A
T
A
G
C
G
T
A
C
A
G
T
C
A
T
G
C
T
C
A
G
C
T
G
A
T
C
A
G
C
A
T
G
C
A
T
G
C
T
G
A
  

|  |  |
| --- | --- |
| p-value: | 1e-12 |
| log p-value: | -2.951e+01 |
| Information Content per bp: | 1.653 |
| Number of Target Sequences with motif | 10.0 |
| Percentage of Target Sequences with motif | 0.85% |
| Number of Background Sequences with motif | 1.4 |
| Percentage of Background Sequences with motif | 0.03% |
| Average Position of motif in Targets | 86.0 +/- 53.3bp |
| Average Position of motif in Background | 107.0 +/- 15.2bp |
| Strand Bias (log2 ratio + to - strand density) | 0.0 |
| Multiplicity (# of sites on avg that occur together) | 1.40 |
| Motif File: | file (matrix) reverse opposite |

### Similar de novo motifs found

|  |  |  |  |  |  |  |  |
| --- | --- | --- | --- | --- | --- | --- | --- |
| Rank | Match Score | Redundant Motif | P-value | log P-value | % of Targets | % of Background | Motif file |
| 1 | 0.620 | A C G T A C G T A G T C A G T C A G T C A G C T A T C G C A T G A C T G A C T G A C T G A G C T | 1e-6 | -14.013172 | 0.60% | 0.06% | motif file (matrix) |
| 2 | 0.657 | A G T C A G T C A G T C A C G T A G C T A G T C A G T C A G T C G A C T A T C G A C T G A C T G A C T G A C G T A C T G C T G A C T A G T A G C A G T C A G T C A G C T A C G T A G T C A G T C A G T C | 1e-2 | -6.218199 | 0.26% | 0.01% | motif file (matrix) |
